# A national survey of the availability of intensity-modulated radiation therapy and stereotactic radiosurgery in Canada

**DOI:** 10.1186/1748-717X-7-18

**Published:** 2012-02-07

**Authors:** Eman Z AlDuhaiby, Stephen Breen, Jean-Pierre Bissonnette, Michael Sharpe, Linda Mayhew, Scott Tyldesley, Derek R Wilke, David C Hodgson

**Affiliations:** 1 Department of Radiation Oncology, University of Toronto, Canada; 2 Radiation Medicine Program, Princess Margaret Hospital, University Health Network, Toronto, Canada; 3 Department of Radiation Oncology, Vancouver Centre, British Columbia Cancer Agency, Vancouver, Canada; 4 Department of Radiation Oncology, Queen Elizabeth II Health Sciences Centre, Dalhousie University, Halifax, Canada; 5 Institute of Health Policy Management and Evaluation, University of Toronto, Canada

**Keywords:** intensity-modulated radiation therapy, stereotactic radiosurgery, survey

## Abstract

**Background:**

The timely and appropriate adoption of new radiation therapy (RT) technologies is a challenge both in terms of providing of optimal patient care and managing health care resources. Relatively little is known regarding the rate at which new RT technologies are adopted in different jurisdictions, and the barriers to implementation of these technologies.

**Methods:**

Surveys were sent to all radiation oncology department heads in Canada regarding the availability of RT equipment from 2006 to 2010. Data were collected concerning the availability and use of Intensity Modulated Radiation Therapy (IMRT) and stereotactic radiosurgery (SRS), and the obstacles to implementation of these technologies.

**Results:**

IMRT was available in 37% of responding centers in 2006, increasing to 87% in 2010. In 2010, 72% of centers reported that IMRT was available for all patients who might benefit, and 37% indicated that they used IMRT for "virtually all" head and neck patients. SRS availability increased from 26% in 2006 to 42.5% in 2010. Eighty-two percent of centers reported that patients had access to SRS either directly or by referral. The main barriers for IMRT implementation included the need to train or hire treatment planning staff, whereas barriers to SRS implementation mostly included the need to purchase and/or upgrade existing planning software and equipment.

**Conclusions:**

The survey showed a growing adoption of IMRT and SRS in Canada, although the latter was available in less than half of responding centers. Barriers to implementation differed for IMRT compared to SRS. Enhancing human resources is an important consideration in the implementation of new RT technologies, due to the multidisciplinary nature of the planning and treatment process.

## Background

Major technical advances have occurred in radiation therapy (RT) over the last two decades. Improvements in RT planning software and the development of dynamic multileaf collimators facilitated the development of Intensity Modulated Radiotherapy (IMRT) [1]. The potential for superior target conformality and reduced dose to normal tissues with IMRT can permit dose escalation which may in turn result in better tumor control without increasing toxicity [2,3]. These dosimetric advantages can produce lower toxicity and possibly better quality of life than seen with non-IMRT treatment [2-9].

Evidence supporting the use of IMRT is mounting [2-13]. Veldeman conducted a systematic review of the clinical evidence for IMRT in 2008 which included 56 comparative studies, 3 of which were randomized controlled trials (RCTs) [11], and concluded that IMRT reduced treatment-related toxic effects and improved quality of life. A second systematic review in 2010 [12] reported reduced acute and late toxicity associated with IMRT [4-10,12,13]. Three RCTs reported significant improvement of acute xerostomia with the use of IMRT in head and neck cancers and better quality of life [6,7,9,13], and IMRT for breast cancer was also associated with reduced acute and late side effects when compared to 2D RT in three RCTs [4,5,8]. With further RCTs in progress [12], additional evidence will soon be available. Other non-randomized studies have similarly shown advantages to IMRT for malignancies in other types of cancer, such as prostate, lung and CNS [10,12,14].

Similarly, stereotactic radiosurgery (SRS) is another comparatively recent innovation in which a single high dose fraction (usually 12 - 24 Gy or higher) is delivered to a target with extreme precision by means of precise immobilization and 3-D planning, allowing for minimal or even no setup margins around the target, thereby minimizing normal tissue damage. Clinical indications for SRS include treatment of brain metastases, meningiomas, acoustic neuromas, and arteriovenous malformations [15]. A 2009 systematic review of the role of SRS in patients with newly diagnosed brain metastases showed a significant survival advantage for patients with single brain metastasis who received SRS following whole brain radiotherapy (WBRT), along with better local control (and possibly survival) for patients with 2-4 brain metastatic lesions [16]. Guidelines regarding the use of SRS for brain metastases have been published in North America [17,18].

Since these technologies were introduced more than a decade ago, there has been a substantial impetus to implement them in clinical practice throughout North America and Europe [19-23]. To our knowledge, there have been few studies evaluating the availability of these technologies in developed countries [19-24]. The purpose of this article is to report the results of a series of surveys across Canada to determine the proportion of cancer centers that provided IMRT and SRS, how these technologies were being used, and the obstacles that hindered their implementation.

## Methods

This survey was part of a study concerning workload and equipment in Canadian radiation oncology centers sponsored by Canadian Association of Radiation Oncology (CARO). From 2006 to 2010, invitations to complete four surveys were sent to Radiation Oncology department heads of all cancer centers across Canada. For each survey, reminders were sent electronically on two separate occasions prior to survey closure. The survey was initially conducted in 2006 then repeated in 2008, 2009, and 2010. The survey was not conducted in 2007 because of concerns about losing respondent buy-in, and so that more detailed questions about the use IMRT and SRS could be integrated into the questionnaire. The survey included 38 cancer centers in 2006 and 2008. One new center was added in 2009 and another in 2010, bringing the total number of surveyed centers to 40 (Table 1). The most recent survey closed in September 2010.

**Table 1:** Response rates across provinces in Canada.

	2006	2008	2009	2010
BC	4/4	4/4	5/5	5/5
Prairies	5/5	5/5	4/5	5/5
ON	12/13	13/13	13/13	14/14
QC	5/10	9/10	8/10	6/10
Atlantic	6/6	6/6	4/6	5/6
Total	32/38 (84%)	37/38 (97%)	34/39 (87%)	35/40 (88%)

*Abbreviations*: BC: British Columbia, Prairies include: Alberta, Saskatchewan, and Manitoba, ON: Ontario, QC: Quebec, Atlantic include: New Brunswick, Nova Scotia, Prince Edward Island, and Newfoundland.

In 2006 and 2008, thirty-eight centers were surveyed. A new cancer center in BC was added in 2009 and another cancer center in ON in 2010.

For the purposes of the survey, IMRT was defined based on the US Medicare definition: created by inverse planning, and one of the following: a) static segmented beams with an average of 5 segments per field, or b) MLC with individually designed compensators (sliding window or intensity modulated arc therapy). When available, Tomotherapy™ was categorized with IMRT. SRS was defined as convergent-beam irradiation that delivers a high dose of radiation to a small target volume within the cranium, delivers the total dose in a single fraction with rapid dose fall-off at the target boundary, and uses either a frame-based or frameless system for target localization.

Included in the survey were questions concerning the availability of computed tomography (CT) simulators, numbers of linear accelerator units (LINAC), capacity to deliver IMRT and SRS, and the utilization of IMRT for specific indications (head and neck, prostate, and breast cancers). Data concerning the provision of SRS, or the existence of a referral system in case of SRS unavailability, were also collected. If a center did not respond to a survey one year however had responded in previous years, we assumed that the equipment had not been reduced, and equipment information from the previous year was used. In some cases, direct communication (telephone/email) correspondence with department respondents was used to clarify missing data. Respondents were asked to indicate if any of nine barriers to implementing IMRT or SRS applied to their center, such as training, equipment configuration, and staffing availability. Participants were also given the option "other".

## Results

The response rates are summarized in table 1. Annual response rates varied from 84% in 2006 to 97% in 2008. The absolute number of reported LINAC units increased from 169 in 2006 to 213 in 2010. The number of LINACs per 10,000 incident cancer patients was similar across provinces and ranged from 14.1 to 16.8. The proportion of LINACs used for IMRT increased from 23.8% in 2006 to 63.2% in 2010. In addition, CT simulation unit numbers have also grown from a total of 40 in 2006 to 57 in 2010. CT simulation units per 10,000 incident cancer patients in 2010 ranged from 2.6 to 4.5 across provinces.

### IMRT Availability

**Figure 1:**
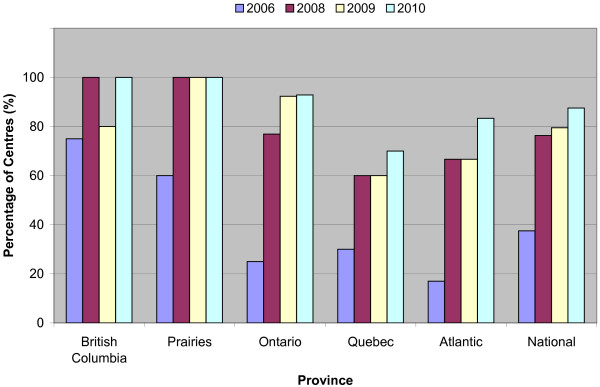
**IMRT availability across Canada**. Within the province of BC, there was a transient drop in 2009 as compared to 2008 as a result of a single center temporarily suspending IMRT during an upgrade of their planning software.

There has been a significant rise in the proportion of centers able to provide IMRT from 2006 to 2010 across all provinces (Figure 1). On a national level, only 37% of centers provided IMRT in 2006, however this percentage increased to 87.5% in 2010. In 2008, 47% of centers indicated that IMRT was available for all patients with a clinical indication, compared to 73% centers in 2010.

**Figure 2:**
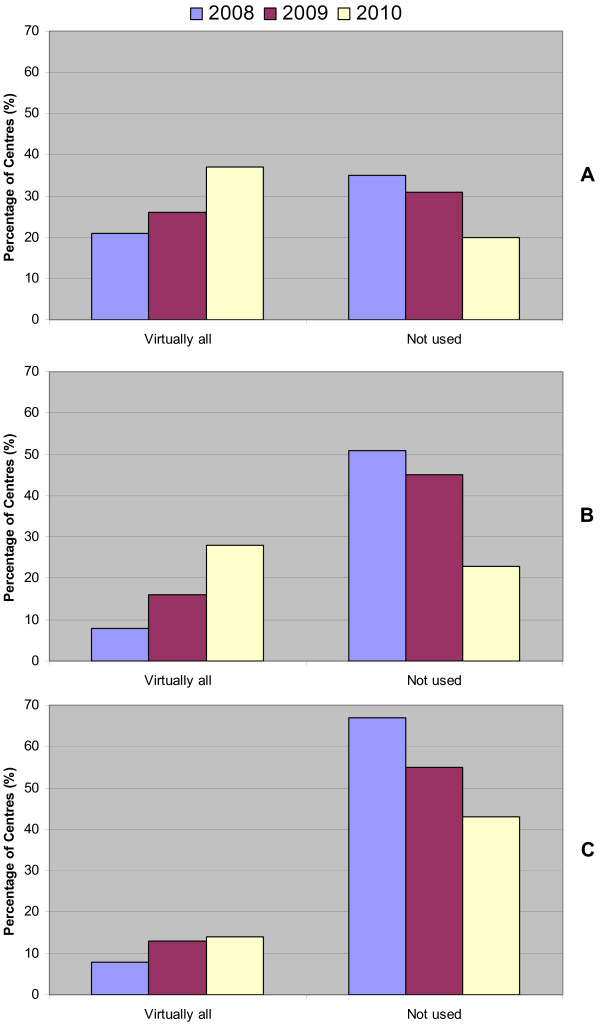
**Site-specific IMRT utilization**. (A) shows IMRT utilization for head and neck cancer patients across cancer centers in Canada. IMRT was provided to "virtually all" patients versus "not used". (B) shows IMRT utilization for prostate cancer patients, and (C) shows IMRT utilization for breast cancer patients.

Corresponding to the increase in the overall availability of IMRT, disease site-specific utilization of IMRT also increased. For example, 65% of cancer centers used IMRT for head and neck cancer patients in 2008, compared to 80% in 2010, and the proportion of centers reporting that IMRT was used for "virtually all" head and neck cases rose from 21% in 2008 to 37% in 2010 (Figure 2A). Similarly, IMRT utilization for "virtually all" patients with prostate cancer increased from 8% in 2008 to 28% in 2010, and breast cancer increased from 8% in 2008 to 14% in 2010 (Figures 2B and 2C). There were corresponding reductions in the proportion of centers indicating that IMRT was not used for these tumor sites.

### Barriers to implementing IMRT

Table 2 shows the results of the surveys concerning IMRT barriers. Among those centers that indicated a barrier to the implementation of IMRT, the most commonly cited barriers were related to recruitment or training of skilled personnel. In the 2010 survey, the most common barriers to IMRT implementation were the need to train existing planners to plan IMRT (50%) and the need to hire more planners (30%). Only a minority of centers indicated that limitations in CT simulator (10%) or linear accelerator configurations (10%) impaired IMRT implementation; however 40% indicated other barriers to implementing IMRT.

**Table 2:** Barriers to IMRT implementation

Barrier to IMRT	2006 (%)	2008 (%)	2009 (%)	2010 (%)
Need to hire more planners	27.3	50	46.7	30
Need to train existing planners to plan IMRT	40.9	72.2	60	50
Need to hire more physicists	40.9	22.2	6.7	10
Need to train existing physicists to plan IMRT	45.5	50	26.7	20
Need to hire more staff to conduct needed QA checks	27.3	N/A	N/A	N/A
Need to hire more oncologists	13.6	16.7	13.3	0
Need to train existing oncologists to deliver IMRT	40.9	44.4	26.7	20
Need to purchase and/or upgrade planning systems	40.9	22.2	20	0
Need to purchase and/or upgrade linear accelerators	27.3	44.4	40	10
Need to purchase and/or upgrade CT simulator	0	22.2	6.67	10
Other	22.7	27.8	40	40

N/A: Data on this barrier was not collected in 2008-2010 surveys

There is a noticeable trend of personnel training-related barriers becoming less prominent over the years. For example, the need to train existing physicists to plan IMRT decreased from 45.5% in 2006 to 20% in 2010. This is also evident for training existing oncologists and planners in addition to hiring more personnel who are trained to perform IMRT planning and treatment.

### SRS availability

**Figure 3:**
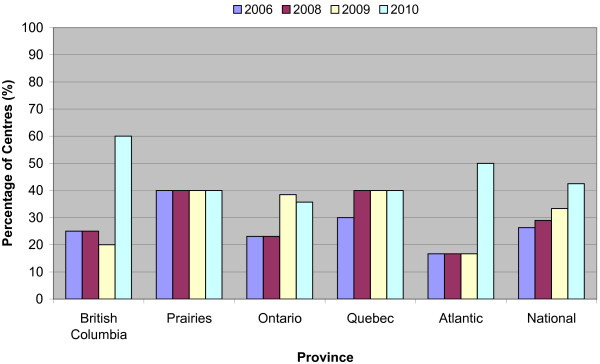
**SRS availability across Canada**. There were decreases in BC and ON as a result of newly established cancer centers that has not implemented the technology.

Overall, SRS became more widely available from 2006 to 2010 although its growth did not equal that of IMRT (Figure 3). SRS was available in 26% of centers in 2006, compared to 42.5% in 2010. Most of the centers (82.6%) that did not perform SRS themselves reported that they had established a referral system for patients who were candidates for SRS treatment. In 2010, 82.5% of centers, including 20 centers that did not have on-site SRS, indicated that patients who might benefit from SRS had access to it either directly or by referral.

### Barriers to implementing SRS

Among those centers that indicated a barrier to the implementation of SRS, implementation was hindered primarily by need to upgrade LINACs or Gamma Knife equipment (83%) as shown in Table 3. Other obstacles included the need to train planners to plan SRS and the need to train more oncologists (50%). Less common barriers included the need to hire more planners and physicists. Barriers mentioned under "other" included lack of a neurosurgery staff and obstacles relating to patient travel to centers where SRS is available. Unlike IMRT implementation barriers, SRS barriers tended to be stable or increased.

**Table 3:** Barriers to SRS implementation

Barrier to SRS	2006 (%)	2008 (%)	2009 (%)	2010 (%)
Need to hire more planners	20	16.7	38	17
Need to train existing planners to plan SRS	20	33.3	38	50
Need to hire more physicists	30	16.7	12.5	33
Need to train existing physicists to plan SRS	20	33.3	12.5	17
Need to hire more staff to conduct needed QA checks	20	N/A	N/A	N/A
Need to hire more oncologists	20	8.3	12.5	17
Need to train existing oncologists to deliver SRS	20	41.7	37.5	50
Need to purchase and/or upgrade planning systems	20	66.7	12.5	33
Need to purchase and/or upgrade LINAC or GK	50	66.7	50	83
Need to purchase and/or upgrade CT simulator	30	16.7	0	0
Other	30	8.3	12.5	17

N/A: Data on this barrier was not collected in 2008-2010 surveys

## Discussion

The adoption and implementation of new radiation therapy technologies has been a challenge for jurisdictions where public health care systems must also contain costs and manage wait times [25]. There are very few data regarding the availability and use of these technologies in different countries. Our data show a rapid implementation of IMRT across Canada from 2006 to 2010. This holds true for the specific cancer disease sites targeted in our survey, with increased utilization of IMRT for head and neck, prostate, and to a lesser extent, breast cancers.

Implementation of IMRT is not without challenges however; particularly early in its implementation, IMRT can require more human resources and is more time consuming to plan than 2D or 3D conformal radiotherapy [26,27]. IMRT also requires a number of supplementary steps for machine commissioning, plan generation and quality assurance (QA). There is a large body of evidence providing detailed descriptions of the QA processes suggested by a number of organizations including American Society of Therapeutic Radiation Oncology (ASTRO) and American College of Radiology (ACR) [28], American Association of Physicists in Medicine (AAPM) [27], European Society of Therapeutic Radiation Oncology (ESTRO) [29], and Cancer Care Ontario (CCO) [30]. The resulting standards regarding the implementation of IMRT programs - practice setting requirements; tools, devices and equipment requirements; professional training requirements; role of personnel; and requirements for quality assurance and safety - all require sufficient staff. If these elements are not available, (and our results suggest that they may be lacking in some Canadian cancer centers), full implementation of IMRT may be limited. The difficulties surrounding IMRT implementation in some centers or jurisdictions may be related to an inadequate understanding of the need to provide funding for additional staffing and training programs in addition to purchasing equipment, and the diversion of existing human resources towards maintaining high patient volumes [27,31].

In 2010, expert panels convened by Cancer Care Ontario provided guideline recommendations with respect to the clinical use of IMRT for a number of cancer sites [32], stating that there was adequate evidence to support the use of IMRT for treatment of head and neck [33] and prostate cancers [34]. IMRT was also recommended for cancers of the breast, lung, central nervous system, and gastrointestinal system in some circumstances [32,35]. Ideally, these guidelines will facilitate appropriate uptake and utilization of IMRT in Canada's largest province, and possibly elsewhere.

Availability of SRS is still limited in Canada with fewer than half of centers providing the treatment in 2010. It is possible that its availability has not grown as rapidly as IMRT owing to the more limited indications for its use, the smaller number of patients who would benefit, and the relatively greater technical requirements. This also may explain why personnel trained to perform SRS are less abundant than those trained to perform IMRT. Moreover, the capital equipment purchase required to develop an SRS program appeared to be a more significant barrier than was described for IMRT. Interestingly, most centers that did not offer SRS indicated that they had an established referral system for eligible patients, although it is not clear how effectively such referral systems work for patients in these centers.

Our data is the first published report of the availability of IMRT and SRS technology in Canada and sheds some light on the factors that hinder its full implementation. Corresponding surveys conducted in the UK reported an increase in the number of centers providing IMRT from 45.8% in 2007 [19] to 76% in 2010 [20]. Obstacles to full implementation included a shortage of planning staff, in addition to a lack of specific funding as IMRT was carried out for the same remuneration as conventional treatment. IMRT appears to have been more rapidly taken up in the United States, with one study finding that 73.2% of radiation oncologists reported using IMRT in 2004 [21] as compared to 32% in 2002 [22]. Reasons to adopt IMRT reported in that survey included the ability to spare normal tissue and dose escalation, in addition to economic competition. Similarly, data from Japan show increased utilization of IMRT in most recent report by Japanese Society of Therapeutic Radiology and Oncology (JASTRO) [24]. These studies can facilitate a more rational approach to health care planning moving forward: new RT technologies will be continually emerging, and these studies demonstrate the multiple factors other than clinical need that contribute to patients access to these technologies.

## Conclusions

There are accumulating data documenting the benefits of IMRT and SRS for reducing the treatment toxicity and improving disease control in selected patients. While IMRT and SRS availability is increasing in Canada, improvement is still required to ensure that everyone who can benefit from these technologies potentially has access to them. Due to the multidisciplinary nature of RT planning and delivery, investment in human resources is an important requirement for adoption and implementation of new RT technologies.

## Competing interests

The authors declare that they have no competing interests.

## Authors' contributions

DH led the survey, and coauthored the manuscript. EZA drafted the manuscript. JPB, MS and SB participated in the survey design, data interpretation, and coauthored the manuscript. LM coauthored the manuscript. ST and DW assisted in collecting survey data, data interpretation, and coauthored the manuscript. All authors read and approved the final manuscript.
